# Adaptive and Innate Immune Responsiveness to *Borrelia burgdorferi sensu lato* in Exposed Asymptomatic Children and Children with Previous Clinical Lyme Borreliosis

**DOI:** 10.1155/2012/294587

**Published:** 2011-12-05

**Authors:** Barbro H. Skogman, Sandra Hellberg, Christina Ekerfelt, Maria C. Jenmalm, Pia Forsberg, Johnny Ludvigsson, Sven Bergström, Jan Ernerudh

**Affiliations:** ^1^Department of Pediatrics, Falun General Hospital, 791 82 Falun, Sweden; ^2^Centre for Clinical Research in Dalarna, Nissers väg 3, 791 82 Falun, Sweden; ^3^Division of Clinical Immunology, Department of Clinical and Experimental Medicine, Linköping University, 581 85 Linköping, Sweden; ^4^Division of Infectious Diseases, Department of Clinical and Experimental Medicine, Linköping University, 581 85 Linköping, Sweden; ^5^Division of Pediatrics, Department of Clinical and Experimental Medicine, Linköping University, 581 85 Linköping, Sweden; ^6^Department of Molecular Biology, Umeå University, 901 87 Umeå, Sweden

## Abstract

Why some individuals develop clinical manifestations in Lyme borreliosis (LB) while others remain asymptomatic is largely unknown. Therefore, we wanted to investigate adaptive and innate immune responsiveness to *Borrelia burgdorferi sensu lato* in exposed *Borrelia*-antibody-positive asymptomatic children (*n* = 20), children with previous clinical LB (*n* = 24), and controls (*n* = 20). Blood samples were analyzed for *Borrelia*-specific interferon (IFN)-*γ*, interleukin (IL)-4, and IL-17 secretion by ELISPOT and *Borrelia*-induced IL-1*β*, IL-6, IL-10, IL-12(p70), and tumor necrosis factor (TNF) secretion by Luminex. We found no significant differences in cytokine secretion between groups, but a tendency towards an increased spontaneous secretion of IL-6 was found among children with previous clinical LB. In conclusion, the adaptive or innate immune responsiveness to *Borrelia burgdorferi sensu lato* was similar in *Borrelia*-exposed asymptomatic children and children with previous clinical LB. Thus, the immunological mechanisms of importance for eradicating the spirochete effectively without developing clinical manifestations of LB remain unknown.

## 1. Introduction

Lyme Borreliosis (LB), caused by the spirochete* Borrelia *(*B.*)* burgdorferi*, is the most common tick-borne infection in both Europe and the USA [1, 2]. The infection may lead to a variety of symptoms by affecting different organs such as the skin, joints, heart muscle, or nervous system. The most common manifestation of LB is the migrating red skin lesion called erythema migrans (EM). LB in children follows a slightly different clinical course than in adults, and duration of symptoms is often shorter [3]. Children seem to have a better prognosis than adults and more seldom report persisting symptoms [4–6]. 

The cells of the innate immune system, constituting the first line of defense, recognize pathogen-associated molecular patterns (PAMPs) through pattern-recognition receptors (PRRs) like Toll-like receptors (TLRs) [7]. The spirochete *B. burgdorferi *contains a high proportion of lipoproteins that are mainly recognized by TLR2 [8]. Recognition of *B. burgdorferi *leads to the release of inflammatory mediators like interleukin (IL)-1*β*, IL-6, IL-10, and IL-12, and tumor necrosis factor (TNF) from monocytes, macrophages, neutrophils, and DCs [9–12]. These cytokines are important for recruitment of other components of the innate host immune response but also for signaling with the adaptive immune system [12]. The adaptive immune system consists of T and B lymphocytes and comprises the second line of defense to eliminate the spirochete. Activated T helper (Th) cells differentiate into Th1, Th2, Th17 or T-regulatory cells [13]. Th1 cells are important for immunity against intracellular pathogens, whereas Th2 cells are involved in immune responses against extracellular parasites [14]. Typical Th1 and Th2 effector cells are macrophages and mast cells, respectively. Th1 and Th2 are defined by their signature cytokines IFN-*γ* and IL-4, which act antagonistically to counterbalance each other. Th17 cells, producing IL-17, are involved in the defense against fungi and some extracellular bacteria [15]. The immune response to *B. burgdorferi *involves both humoral and cell-mediated immune responses where both T-cell-independent and -dependent B cell responses are important during the adaptive immune response for killing the spirochetes [12]. 

Children and adults differ in the type of immune response they evoke when encountering *B. burgdorferi. *In adults, the immune response in LB is characterized by a strong Th1 responses with high numbers of *Borrelia-*specific IFN-*γ*-secreting cells and low levels of IL-4 [16, 17], whereas children seem to have a more balanced immune response with elevated secretion of both IFN-*γ* and IL-4 [18]. It has been hypothesized that the type of immune response evoked in the presence of the *Borrelia *spirochete may have significant effect on the clinical course and the outcome of the infection [19]. Persistent symptoms after LB were associated with a strong IFN-*γ* response but lacked the subsequent upregulation of IL-4 [19]. Thus, it appears that a later prominent Th2 immune response is necessary to downregulate the initial strong IFN-*γ* response in order to effectively terminate the infection and hinder unsatisfactory tissue damage. This could be one possible explanation why children usually experience a more benign course of the disease as they show both strong Th1 and Th2 immune responses [18]. 

The fact that some individuals may be exposed to *B. burgdorferi s.l. *without developing clinical symptoms is interesting from an immunological standpoint and could indicate a more effective immune response to the spirochete in these individuals. In adults, the term asymptomatic *Borrelia *infection is used for individuals who have been exposed to *B. burgdorferi s.l. *(i.e., with *Borrelia* IgG antibodies in serum) without known previous clinical LB [20]. These asymptomatic individuals have been found to have a higher secretion of the proinflammatory cytokines IL-12 and TNF than patients with clinical LB, suggesting an enhanced innate activity [21]. As for the adaptive immune responses, no differences have been found in *Borrelia-*specific IFN-*γ* and IL-4 secretion when comparing *Borrelia* exposed asymptomatic adults to patients with clinical LB [20]. To our knowledge, the innate and adaptive immune responses have not previously been studied in *Borrelia* exposed asymptomatic children. 

The aim of this study was to investigate adaptive and innate immune responsiveness to *Borrelia *exposed asymptomatic children as compared to children with previous clinical LB to elucidate immunological mechanisms that might contribute to an effective eradication of the pathogen.

## 2. Materials and Methods

Children recruited to participate in this study were initially included in a larger prospective study, the ABIS (All Babies In Southeast Sweden) study, with the primary purpose of finding risk factors for immune-mediated diseases, mainly Type 1 diabetes (*n* = 17  055). These children were followed until at 5 years of age at primary health care centers. All participating families completed a validated questionnaire, and venous or capillary blood samples were collected in conjunction with the 5-year followup. Two thousand children were randomly selected and screened for *Borrelia *IgG antibodies in serum [22]. Information concerning gender, geographic location, known tick bites, previous LB, and antibiotic treatment for LB was collected from the questionnaire. 

Of these 2000 children, a total number of 64 children, geographically spread, were chosen to represent three major groups: *Borrelia* exposed asymptomatic children (*n* = 20), children with previous clinical LB (*n* = 24), and controls (*n* = 20) (Table 1). *Borrelia* exposed asymptomatic children were characterized by having *Borrelia *IgG antibodies in serum, but they reported no previous symptoms or treatment for LB in the questionnaire. Children with previous clinical LB reported previous treatment for LB, and some of them had *Borrelia* antibodies in serum (4/24). The control group reported no symptoms or previous treatment for LB and had no *Borrelia *antibodies in serum (Table 1). The control group and children with previous clinical LB were matched for gender and geographic location with *Borrelia* exposed asymptomatic children (Table 1). All 64 children were included for analysis with the ELISPOT assay but some later had to be excluded (*n* = 16) due to low responses in postive controls (see *Data Handling*). These excluded children did not differ statistically concerning gender (female 7/16 versus 22/48) or geographic location (rural living 7/16 versus 19/48) compared to included children (*n* = 48). Samples from 41 children were used for analysis with Luminex due to insufficiencies in cell samples (see *Data Handling*). The excluded children (*n* = 23) did not differ statistically concerning gender (female 10/23 versus 19/41) or geografic location (rural living 10/23 versus 16/41) compared to included children (*n* = 41). Informed consent has been given by all participating families, and the study was approved by the Regional Ethical Committee at the Faculty of Health Sciences, Linköping, Sweden (Dnr 03-547). All laboratory work in this study (i.e., not the collection and cryopreservation of cells) has been carried out by one person (S. Hellberg, one of the authors).

### 2.1. ELISA Antibody Test for *B. burgdorferi s.l.*

A commercial enzyme-linked immunoassay (ELISA) kit, based on the *Borrelia*-specific protein Flagellin, was used (IDEA *Borrelia burgdorferi* IgG kit, DakoCytomation, Glostrup, Denmark and Oxoid Limited, Hampshire, United Kingdom) [24], and cut-off for OD values was set according to the manufacturer's instructions.

### 2.2. Isolation, Cryopreservation, and Thawing of Peripheral Blood Mononuclear Cells (PBMCs)

Blood samples were collected from the primary health centers, sent to the Division of Pediatrics, Linköping University, and prepared for isolation and cryopreservation as described in earlier studies [25]. When the time came for analyses, the samples were taken out of the nitrogen container and thawed in a 37°C water bath. Once thawed, the cell suspension was immediately transferred into a 15 mL polypropylene tube, and prewarmed (37°C) tissue culture media (TCM) containing 10% heat-inactivated fetal calf serum (FCS; Sigma Aldrich, Stockholm, Sweden) and Iscove's modification of Dulbecco's medium (GIBCO, Paisley, UK) supplemented with L-glutamine (Sigma Aldrich Sweden AB, Stockholm, Sweden) 292 mg L^−1^, MEM (minimum essential media) 100 X nonessential amino acids 10 *μ*g mL^−1^ (Invitrogen AB, Paisley, UK), penicillin 50 IU mL^−1^, streptomycin 50 *μ*g mL^−1^ (BioWhittaker Europe, Essen, Germany), and NaHCO_3 _3.024 g L^−1^ (Merck KGaA, Damstedt, Germany) were added dropwise along the wall of the tube to avoid osmotic shock in the cells. The cell suspension was centrifuged for 10 minutes at 400 ×g at room temperature and the supernatant discarded. The cells were washed twice in TCM at 400 ×g at RT for 10 min. The cells were counted using a Bürker chamber in a phase-contrast microscope (Carl Zeiss AB, Stockholm, Sweden). The cell membrane integrity, that is, viability of the cells, was assessed with trypan blue exclusion and ranged from 83% to 99% with a median of 95%. The concentration of cells was adjusted to 1 × 10^6^ PBMC mL^−1^.

### 2.3. Preparation of the *Borrelia* Outer Surface Protein-Enriched Fraction (OF) Antigen

The cells, both in ELISPOT assay and *in vitro* stimulation for Luminex assay, were stimulated by OF, consisting primarily of OspA and OspB from *B. garinii *strain Ip90. This antigen was chosen because it has previously been shown to be effective in differentiating between individuals with *Borrelia *infections and controls in ELISPOT assay for specific *Borrelia *IFN-*γ* and IL-4 secretion [17, 23]. The antigen was prepared as described in earlier reports [26, 27], and the optimal concentration of the *Borrelia *OF antigen was determined through testing of different antigen concentrations (54, 18, 6, and 2 *μ*g mL^−1^) on an adult patient sample with confirmed neuroborreliosis (NB) by ELISPOT analysis. The final optimal concentration of *Borrelia *OF antigen was therefore 6 *μ*g/mL^−1^ in the ELISPOT assay. In the Luminex assay, a concentration of 12 *μ*g/mL was used.

### 2.4. ELISPOT Analysis for IFN-*γ*, IL-4, and IL-17

To determine the T-cell response to *B. burgdorferi s.l., *an ELISPOT assay was used to assess the number of *Borrelia-*specific IFN-*γ*-, IL-4- and IL-17-secreting cells. The ELISPOT analyses, originally described by Czerkinsky et al. [28], were performed according to the instructions provided by the manufacturer (Mabtech AB, Nacka, Sweden) and as described in detail in earlier studies [17, 23]. Tetanus toxoid (TT; Swedish Institute for Infectious Disease Control, Stockholm, Sweden) and phytohemagglutinin A (PHA; Sigma-Aldrich AB, Stockholm, Sweden) were used as positive controls, representing recall antigen and polyclonal stimulation, respectively, at a final concentration of 5 LF units mL^−1^ for TT and 20 *μ*g mL^−1^ for PHA. A peptide pool consisting of 32 peptides derived from the human Cytomegalovirus, Epstein-Barr virus, and Influenza virus (CEF; Mabtech AB, Nacka, Sweden) was also included as an additional positive control for IFN-*γ* responses and used at a final concentration of 2 *μ*g mL^−1^. As for negative controls, wells with TCM were used without cells. All samples (except wells containing only PHA or cell medium alone) were assayed in triplicate although some samples could only be assayed in duplicate due to low cell count. The spots were counted manually by dissection microscope and by semiautomatic AID EliSpot Reader system/HR version 3.2.3 (AID autoimmune diagnostics GmbH, Strassberg, Germany) in a blinded manner on one single occasion by the same person (S. Hellberg, one of the authors). Each spot counted represented one cytokine-producing cell.

### 2.5. Luminex Analysis for IL-1*β*, IL-6, IL-10, IL-12(p70), and TNF

Half a million PMBCs diluted in 0.5 mL TCM supplemented with 10% FCS (Sigma Aldrich, Stockholm, Sweden) were cultured together with OF from *B. garinii* and LPS (Sigma-Aldrich AB, Stockholm, Sweden) from *Salmonella typhimurium *at final concentrations of 12 *μ*g mL^−1^, 1 ng mL^−1^, and 100 ng mL^−1^, respectively, or without antigen, at 37°C, 5% CO_2_, for 24 hours. After 24 hours, the cells were centrifuged (400 ×g at RT), and the supernatants were collected and frozen at −70°C until used for the Luminex analysis of cytokines in the samples. The levels of cytokines IL-1*β*, IL-6, IL-10, IL-12(p70), and TNF were measured in the PBMC supernatants by a Bio-Plex Pro Human Cytokine Panel Kit (Bio-Rad Laboratories, Calif, USA). All assays were carried out in accordance with the instructions provided by the manufacturer. The plates were then analyzed using Luminex 200 (Invitrogen, Merelbeke, Belgium). The analysis condition was set to a minimum of 100 beads per region. The raw data, median fluorescent intensity (MFI), was analyzed using xPONENT 3.1 (Luminex Corporation, Austin, Tex, USA). The quantifiable ranges for the standard curves were for IL-1*β* 1.91–1959.31 pg mL^−1^, IL-6 1.54–25171 pg mL^−1^, IL-10 1.48–6076 pg mL^−1^, TNF 4.75–19438.75 pg mL^−1^, and for IL-12(p70) 2.19–8988 pg mL^−1^. Values below the lowest detection limit of the standard curve were assigned half the value of the detection limit, and values above highest detection limit were assigned double the value of the detection limit.

### 2.6. Data Handling

Regarding the ELISPOT assay, the median of the triplicates or duplicates was used for the analysis of cytokine-secreting cells. The method for determining *Borrelia-*specific secretion in this study has previously been implemented in other studies [18–20] and is based on both the unstimulated, spontaneous secretion and the antigen-stimulated secretion of the cells. The *Borrelia-*specific secretion was determined by subtracting the number of spots from the wells with cell suspension and medium (i.e., the spontaneous secretion) from the number of spots in the OF-antigen-stimulated wells (i.e., *Borrelia-*stimulated secretion) [20]. Both the *Borrelia*-specific secretion and the spontaneous secretion are interesting to show to give a full picture of immune responses, and both are therefore reported in Results, Discussion, and Figures. 

All 64 samples were investigated by the ELISPOT assay. However, as there were low responses in some of the positive controls, defined criteria were used to assure that cells had the ability to respond. These criteria were mainly based on the PHA responses for IFN-*γ*. Samples with a high PHA response for IFN-*γ* (over 300 spots) were all included (*n* = 27). Samples with PHA response of 200–300 spots were included if they also had an apparent antigen-induced response for TT and CEF (for more than one cytokine) or a strong PHA response for both IL-4 and IL-17 (*n* = 19). Based on previous experience, samples with a PHA response for IFN-*γ* showing less than 200 spots were excluded (*n* = 16) with the exception of two samples showing strong antigen-induced responses for both TT and CEF (for more than two cytokines) and strong PHA responses for both IL-4 and IL-17 (*n* = 2). All these considerations were blinded from the belonging to groups and the *Borrelia-*stimulated secretion. 

With regard to the Luminex assay, 41 samples were available and all were analyzed. The ability of the cells to respond to stimuli was assessed by the ratio between the samples stimulated with LPS and the spontaneous secretion. Samples were included if they had an LPS/spontaneous secretion ratio of five or more for any of the analyzed cytokines. Based on these criteria, all samples were valid and could be included for analysis (*n* = 41). The *Borrelia* induced secretion was obtained by subtracting spontaneous secretion from the samples stimulated with *Borrelia* OF antigen as previously implemented for the ELISPOT assay.

### 2.7. Statistical Analysis

Statistical Products and Service Solutions (SPSS), version 17.0 for Windows was used for the statistical analysis. The Kruskal-Wallis test was used as a pretest for comparison of the immunological parameters between the groups. Mann-Whitney *U* test was used as a post hoc test when the *P* value for Kruskal-Wallis test was *P* < 0.08. For Mann-Whitney, a level of *P* < 0.05 was considered statistically significant. Since the cytokines analyzed in this study were viewed as part of a pattern and not as separate events, no corrections for multiple comparisons were made.

## 3. Results

### 3.1. Number of IFN-*γ*-, IL-4- and IL-17- Secreting Cells Measured by ELISPOT

**Figure 1:**
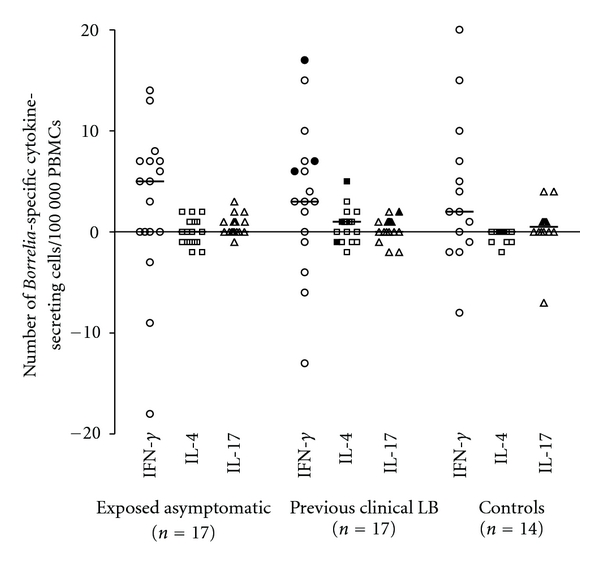
The number of *Borrelia-*specific IFN-*γ*-secreting cells (open circle), IL-4-secreting cells (open square) and IL-17-secreting cells (open triangle), per 100 000 PBMCs as measured by ELISPOT in different groups. The filled circles, squares, and triangles represent children in the previous clinical LB group with *Borrelia* seropositivity. The *Borrelia-*specific secretions are net values obtained after subtracting the number of spontaneous cytokine-secreting cells from the number of outer surface protein fraction (OF-) antigen-specific cytokine-secreting cells. The median values are noted as lines in the figure. No statistically significant differences were found between groups.

All groups displayed a predominance of *Borrelia*-specific IFN-*γ*-secreting cells as compared to IL-4- and IL-17-secreting cells. However, no significant differences were found between *Borrelia* exposed asymptomatic children and children with previous clinical LB regarding the number of *Borrelia-*specific IFN-*γ*-, IL-4-, and IL-17-secreting cells (Figure 1). Moreover, no significant difference could be found in comparison to the control group for any of the analyzed cytokines. Some of the individuals in all three groups had negative values for the *Borrelia-*specific secretion, that is, the spontaneous secretion was higher than the OF-stimulated secretion (Figure 1).

**Figure 2:**
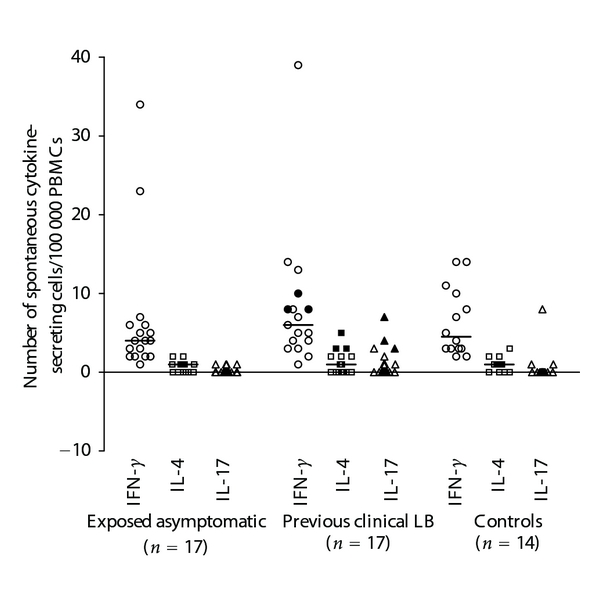
The number of spontaneously IFN-*γ*-secreting cells (open circle), IL-4-secreting cells (open square), and IL-17-secreting cells (open triangle) per 100 000 PBMCs as measured by ELISPOT in different groups. The filled circles, squares, and triangles represent the children in the previous clinical LB group with *Borrelia *seropositivity. The median values are noted as lines in the figure. No statistically significant differences were found between groups.

When analyzing the number of spontaneous secreting cells, indicating the nonstimulated background activity of cytokine secretion, no significant differences in IFN-*γ*, IL-4, or IL-17 was found between any of the groups (Figure 2). A ratio between the numbers of IL-4- and IFN-*γ*- spontaneously secreting cells, assessing the Th2/Th1 balance, did not show any significant differences across groups (data not shown). PHA-induced secretion, indicating the ability of the cells to respond to mitogenic stimulation, elicited a stronger response for IFN-*γ* than for IL-4 and IL-17, but no differences were found between groups (data not shown).

### 3.2. IL-1*β*, IL-6, IL-10, IL-12(p70), and TNF Secretion Measured by Luminex

The *Borrelia* induced secretion of IL-1*β*, IL-6, IL-10, and TNF were readily detectable with the Luminex assay, whereas the *Borrelia* induced secretion of IL-12(p70) was undetectable in all samples (Table 2). Moreover, the IL-12(p70) levels were also below the detection limit also in 32 of the 40 samples stimulated with LPS and did not exceeded 8 pg/mL in the remaining 8 samples. Thus, levels of IL-12(p70) were not considered interpretable. Of the remaining cytokines, IL-6 was present in the overall highest concentration in all groups and IL-10 in the lowest concentration in all groups (Table 2). 

**Figure 3:**
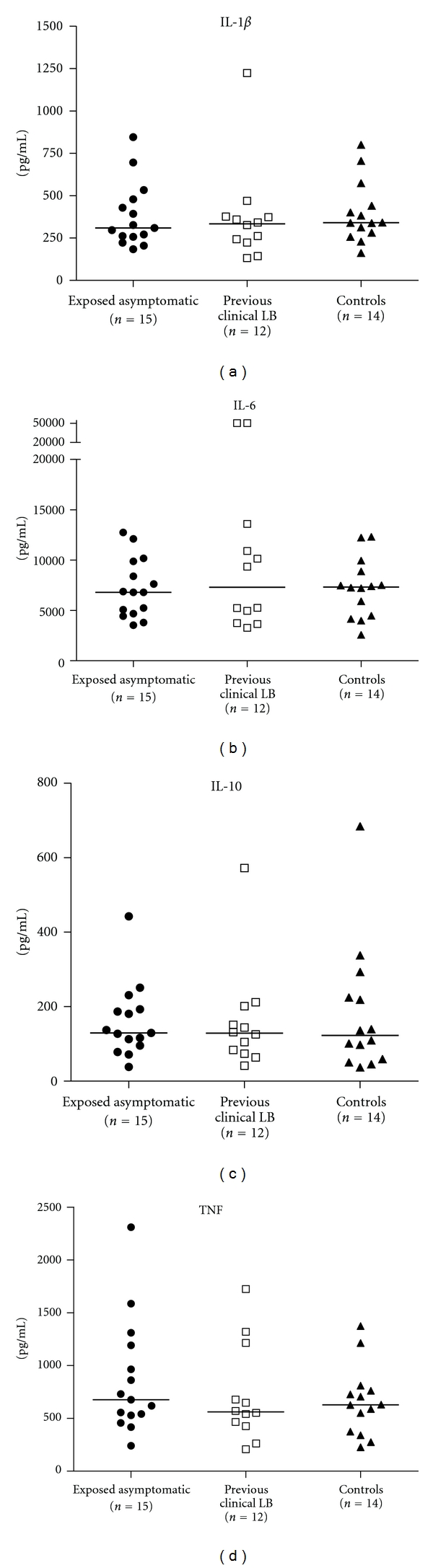
The *Borrelia*-induced secretion of IL-1*β*, IL-6, IL-10, and TNF in PBMC supernatants from *Borrelia* exposed asymptomatic children (filled circle), children with previous clinical LB (open square), and controls (filled triangle) as measured by Luminex. The *Borrelia* induced secretions are net values obtained after subtracting the level of spontaneous cytokine secretion from the level of outer surface protein-fraction (OF-) stimulated cytokine secretion. The median values are noted as lines in the figure. No statistically significant differences were found between groups.

**Figure 4:**
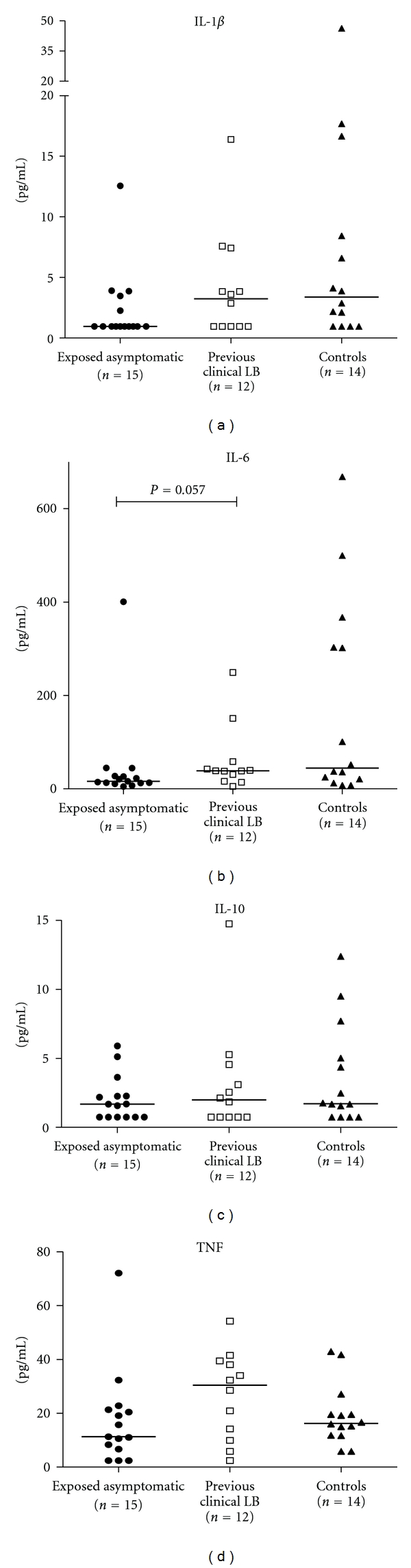
The spontaneous secretion of IL-1*β*, IL-6, IL-10, and TNF in PBMC supernatants from *Borrelia* exposed asymptomatic children (filled circle), children with previous clinical LB (open square), and controls (filled triangle) as measured by Luminex. The median values are noted as lines in the figure. There was a tendency to higher IL-6 in children with previous clinical LB compared to *Borrelia* exposed asymptomatic children, otherwise no statistically significant differences were found between groups.

As for the *Borrelia* induced secretion, no significant differences were found between *Borrelia* exposed asymptomatic children, children with previous clinical LB, and controls with regard to IL-1*β*, IL-6, IL-10, and TNF (Figure 3). The spontaneous secretion did not differ significantly between groups for any of the cytokines although there was a tendency (*P* = 0.057) towards an increased spontaneous secretion of IL-6 in children with previous clinical LB as compared to *Borrelia* exposed asymptomatic children (Figure 4). The ratio between LPS-stimulated secretion and the spontaneous secretion, indicating the ability of the cells to respond to a TLR4-agonistic stimulus, was (median values with range in parenthesis): 301(9–2051) for IL-1*β*; 314(8–4262) for IL-6; 134(17–450) for IL-10 and 66(7–652) for TNF (data not shown).

## 4. Discussion

In this study, we have investigated the *Borrelia-*specific (adaptive) and the *Borrelia* induced (innate) immune responses in *Borrelia *exposed asymptomatic children and children with previous clinical LB. Our aim was to better understand immunological mechanisms that could explain why some individuals develop clinical manifestations of LB and others do not. Interestingly, we found no differences in the number of *Borrelia-*specific IFN-*γ*-, IL-4-, and IL-17-secreting cells when comparing *Borrelia* exposed asymptomatic children, children with previous clinical LB, and controls. This lack of *Borrelia*-specific (adaptive) immune responsiveness is congruent with earlier studies on *Borrelia* exposed asymptomatic adults where no differences in the number of *Borrelia-*specific IFN-*γ*- and IL-4-secreting cells were found compared to patients with clinical LB [20]. Our negative findings are further supported by Jarefors et al. [29], who found no differences in IFN-*γ* secretion between asymptomatic adults and patients with previous clinical LB [29]. As for the innate immune responses, no significant differences in the *Borrelia* induced cytokine IL-1*β*, IL-6, IL-10, or TNF secretion between groups were found in our study, whereas in a previous study by Sjöwall et al. [21], an increased number of *Borrelia*-induced TNF-secreting DCs were found in asymptomatic adults as compared to patients with a history of NB [21]. 

We are well aware of the fact that the number of patients in each group is rather low; but with well-characterized patient groups, a proper design of the study and nonparametric statistic calculations, the results should be reliable. Furthermore, the fact that the number of *Borrelia-*specific IFN-*γ*- and IL-4-secreting cells was generally lower as compared to earlier studies [17, 18, 20] led us to consider that the responsiveness of the cells to stimuli might have been reduced due to the quality of the cells after freezing (previous studies were performed on freshly isolated cells). However, the response to PHA was similar to that obtained from another study on LB in adults [17], and additionally the response to LPS was substantially higher than the spontaneous secretion which, considered together, confirms the ability of the cells to respond to stimuli. The use of cryopreserved PBMCs in this study was necessary due to the design of the study, as it would have been impossible to test cellular immune responses in freshly isolated PBMCs from thousands of unselected children. Earlier reports on cryopreservation effects on cytokine secretion by ELISPOT showed a general decrease in the IL-4 secretion in cryopreserved cells as compared to fresh cells both in spontaneous and allergen-induced secretion, whereas IFN-*γ* secretion was less affected [30]. Thus, to some extent, the cryopreservation could explain the low levels of IL-4-secreting cells found in this study as compared to earlier studies with freshly prepared PBMCs from children (1–17 years old) [18]. It is also important to note that blood samples from children were taken postinfection and not during the actual infection, in accordance with the design of the study.

The young age of the children has also to be taken into consideration. The ability to respond with IFN-*γ* is impaired in neonates [31] and develops during childhood [32, 33]. The children in our study were all 5 years of age, and thus one would expect full capacity to respond with IFN-*γ* to stimuli [34, 35]. A similar capacity was found when comparing the PHA-induced IFN-*γ* secretion in our present study to the PHA-induced IFN-*γ* secretion in adults [17].

Whether or not children with previous clinical LB are “truly” *Borrelia* exposed, patients may be a matter for discussion. Data in our present study is based on self-reported information that may have weaknesses. However, most patients did report a previous EM, which is a clear clinical diagnosis, and although self-reported, the diagnosis was stated to be set by a physician and treated with antibiotics. Very few of the children with previous clinical LB were *Borrelia *seropositive (*n* = 4, Table 1), but this was expected since EM is a clinical diagnosis set by a physician, the sensitivity of the test in EM is low and antibody levels may be reduced after antibiotic treatment [36]. Previous antibiotic treatment in this group could theoretically also have influenced immune responses. Moreover, one must remember that our study mainly evaluates children with previous EM (only 5 children with facial palsy and 1 with meningitis), thus conclusions on immune responses in disseminated LB could not be drawn from our data.

Furthermore, whether or not *Borrelia* exposed asymptomatic children are “truly” *Borrelia* exposed or falsely seropositive may also be a matter for discussion. We have not carried out any confirmatory test since the specificity of the test is high [37], and in earlier studies, a *Borrelia*-specific T-cell response in PBMCs was noted in *Borrelia* exposed asymptomatic adults, certifying a true exposure [38]. Thus, we believe that false seropositive specimens should not be a problem in our material.

We found a tendency towards higher levels of spontaneous secretion of IL-6 (by Luminex) in children with previous clinical LB (Figure 4). IL-6 is a pleiotropic cytokine that mainly mediates proinflammatory effects, and it induces secretion of IL-17 from naïve T cells [39] and may therefore, together with IL-17, be involved in pathogenesis of LB. Recently, it was suggested that IL-17 might contribute to the pathogenesis in Lyme arthritis and in NB [40, 41]. Thus, one could speculate that IL-6 together with IL-17 may be involved in inflammatory mechanisms contributing to clinical manifestations of LB. However, in the present study, we found only a tendency of elevated spontaneous secretion of IL-6 but no *Borrelia* induced IL-6 or IL-17 secretion. This could possibly be explained by the fact that we have analyzed PBMCs after inflammation and not specimen from immune privileged sites during active inflammation. Thus, the role of IL-6 together with IL-17 is certainly interesting regarding the pathogenesis in LB but needs further investigation. 

Finally, why some individuals develop a clinical disease upon encountering *B. burgdorferi s.l. *and some do not remains unclear. Whether or not specific properties of the host's immune system are of importance is still not understood, and, admittedly, there might be other important aspects. For example, the *Borrelia* genospecies infecting the human might play a substantial role in the different clinical outcomes observed in LB. It is well established that the different genospecies of *B. burgdorferi s.l. *can cause different clinical manifestations, and it is also well known that different genospecies have different abilities in escaping the complement system, thereby avoiding elimination [42]. These aspects, as well as the individual genetic predisposition might be crucial factors in understanding mechanisms in the spirochete-host interaction, and future studies are warranted in these fields. 

In conclusion, our results show no differences in adaptive or innate immune responsiveness to *B. burgdorferi s.l. *when comparing *Borrelia* exposed asymptomatic children and children with previous clinical LB. Thus, immunological mechanisms of importance for eradicating the spirochete effectively without developing clinical manifestations of LB remain unknown.

## Figures and Tables

**Table 1:** Subject characteristics at 5 years of age.

	Exposed asymptomatic	Previous clinical LB	Controls
	(*n* = 20)	(*n* = 24)	(*n* = 20)
Gender (f/m)	9/11	11/13	9/11
Rural living	7	11	8
Known tick bites	20	24	0
Previous clinical LB			
EM	0	22	0
NB, facial nerve palsy	0	5	0
NB, meningitis	0	1	0
Antibiotic treatment for LB	0	24	0
*Borrelia* IgG antibodies in serum*	20	4	0

Note. The data referred to in the table is given as numbers of children. Some of the children with previous clinical LB presented with several symptoms. *n*: number; f: female; m: male; LB: Lyme borreliosis; EM: Erythema migrans; NB: Neuroborreliosis; IgG: immunoglobulin G

∗Based on an ELISA (DAKO) kit for IgG antibodies for Borrelia-specific flagella antigen [24].

**Table 2:** The OF induced cytokine secretion in PBMCs by Luminex.

Cytokine	Exposed asymptomatic	Previous clinical LB	Controls
IL-1*β*	309 (183–846)	341 (132–1223)	340 (162–800)
IL-6	6805 (3528–12749)	7304 (3281–50303)	7335 (2578–12324)
IL-10	130 (38–442)	129 (42–572)	123 (37–684)
IL-12(p70)	1 (0-1)	1 (0-1)	1 (0-1)
TNF	676 (241–2311)	561 (209–1724)	628 (226–1373)

NOTE. The data referred to in the table is given as median values in pg/mL (range in parenthesis).

OF: outer surface protein enriched fraction; PBMCs: peripheral blood mononuclear cells; LB: Lyme Borreliosis; IL = interleukin; TNF: tumour necrosis factor.

## List of Abbreviations

ABIS: “All Babies In Southeast Sweden”
*B. burgdorferi s.l*.: *Borrelia burgdorferisensu lato*
CEF: Peptide pool consisting of 32 peptides derived from human Cytomegalovirus, Epstein-Barr Virus, and Influenza virus
CSF: Cerebrospinal fluid
DC: Dendritic cell
ELISA: Enzyme-linked immunosorbent assay
ELISPOT: Enzyme-linked immunospot assay
EM: Erythema migrans
f: Female
FCS: Fetal calf serum
IFN-*γ*: Interferon gamma
Ig: Immunoglobulin
IL: Interleukin
LB: Lyme borreliosis
LPS: Lipopolysaccharides
m: Male
NB: Neuroborreliosis
OF: Outer surface protein-enriched fraction
OD: Optical density
PAMPs: Pathogen-associated molecular patterns
PBMCs: Peripheral blood mononuclear cells
PHA: Phytohaemagglutinin A
PRRs: Pattern recognition receptors
SPSS: Statistical Products and Service Solutions
TCM: Tissue culture medium
Th1: T helper lymphocyte type 1
Th2: T helper lymphocyte type 2
Th17: T helper lymphocyte type 17
TLR: Toll-like receptor
TNF: Tumor necrosis factor
TT: Tetanus toxoid.
